# RegB/A-dependent redox regulation links iron uptake to methanol metabolism in *Methylomonas* sp. DH-1

**DOI:** 10.1128/aem.00495-26

**Published:** 2026-05-19

**Authors:** Wooyoung Park, Seungwoo Cha, Hyeonwoo Mun, Kang-Lok Lee, Sang Woo Seo, Ji-Sook Hahn

**Affiliations:** 1 Department of Chemical and Biological Engineering, Institute of Chemical Processes, Seoul National University 543340, Seoul, Republic of Korea; 2 Department of Biology Education, IALS, Gyeongsang National University 26720, JinJu, Republic of Korea; University of Nebraska-Lincoln, Lincoln, Nebraska, USA

**Keywords:** iron uptake, methanol redox stress, methanotroph, RegB/RegA redox sensor, Na⁺-transporting NADH:ubiquinone reductase

## Abstract

Methanotrophs primarily use methane as a carbon and energy source, but they can also grow on methanol. However, methanol assimilation in the absence of methane oxidation results in a redox imbalance due to the accumulation of NADH. In *Methylomonas* sp. DH-1, a type I methanotroph, we show that this methanol-induced redox stress activates the RegB/RegA two-component system, a global redox sensor. Methanol triggers the RegB-to-RegA phosphotransfer cascade, resulting in RegA-dependent expression of the *feo* operon and increased ferrous iron uptake. This iron acquisition is essential for methanol growth, as it supports the function of Na^+^-transporting NADH:ubiquinone reductase (Na^+^-NQR), an iron-sulfur cluster-containing enzyme required for NADH oxidation. The operon encoding Na^+^-NQR complex is upregulated independently of RegA. Together, these findings reveal a redox-sensitive regulatory mechanism in which the RegB/RegA system senses redox imbalance and promotes iron uptake to facilitate Na^+^-NQR-mediated NADH reoxidation, thereby restoring redox homeostasis. This study uncovers a critical adaptive strategy for maintaining redox balance during methanol metabolism in methanotrophs.

**IMPORTANCE:**

Methanotrophs are emerging as sustainable platforms for converting methane into value-added chemicals. Many species can also utilize methanol—a readily available and industrially attractive alternative—as a carbon source. However, their broader industrial applications have been limited by an incomplete understanding of the fundamental physiological processes that underlie their metabolic and regulatory responses. In this study, we demonstrate how *Methylomonas* sp. DH-1, an aerobic methanotroph, couples carbon source sensing with redox regulation through the conserved RegB/RegA two-component system. We show that RegB/RegA controls iron uptake in response to methanol-induced redox stress, which, in turn, supports NADH reoxidation via Na⁺-NQR. These findings reveal a redox-responsive regulatory mechanism for methanol utilization and provide a foundation for engineering methanotrophs optimized for methane and methanol bioconversion.

## INTRODUCTION

Methane, derived from natural gas and biogas, is a highly competitive substrate for industrial biosynthesis due to its low cost and high reducing power (1). Accordingly, there is growing interest in developing technologies that convert methane into value-added chemicals. However, conventional chemical methods often require high temperatures and significant energy input, primarily because of the high dissociation energy of the first C-H bond cleavage in methane (1, 2). Although methane is already widely used to produce various chemicals and energy carriers, these thermal processes remain energy-intensive compared to biological conversion under mild conditions. Methanotrophs, a specialized group of bacteria that utilize methane as both a carbon and energy source, have emerged as promising biocatalysts for methane conversion. Recent advancements in genetic tools have enabled the successful bioconversion of methane into value-added products, including lactate (3), ectoine (4), cadaverine (5), succinate (6), polyhydroxyalkanoate (7), and acetol (1, 8–10).

**Fig 1:**
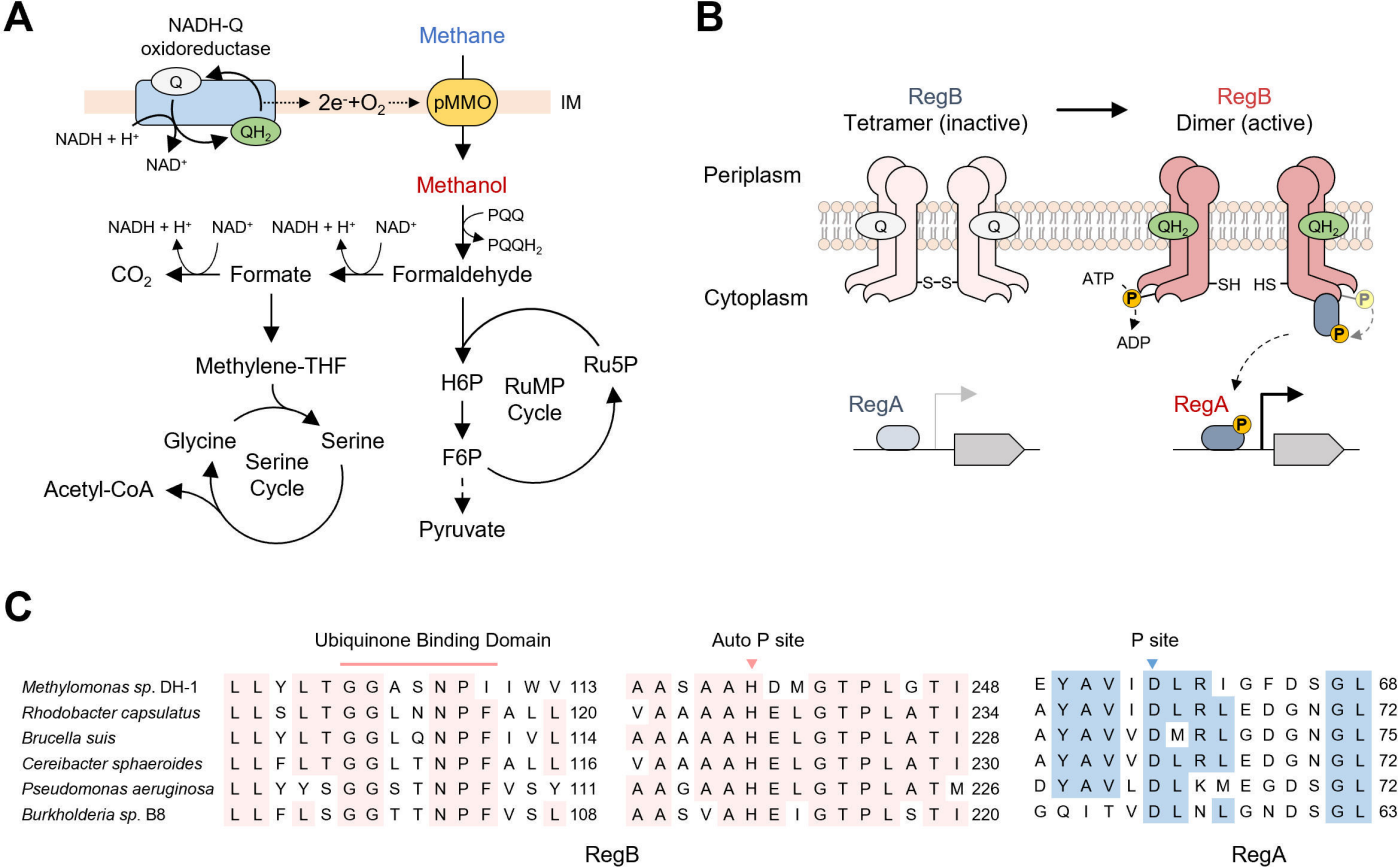
Global redox regulating RegB/A system in *Methylomonas* sp. DH-1. (**A**) Methane metabolism of *Methylomonas* sp. DH-1, pMMO, particulate methane monooxygenase; H6P, hexulose-6-P; F6P, fructose-6-P; Ru5P, ribulose-5-P; Methylene-THF, methylenetetrahydrofolate. (**B**) Schematic illustration of the RegB/A system and its redox-sensing regulatory mechanisms. (**C**) Sequence homology alignment of the RegB/A system in *Methylomonas* sp. DH-1 and other representative bacteria, P site, phosphorylation site.

Methane oxidation in methanotrophs is initiated by methane monooxygenases (MMOs), which exist in two forms: soluble MMO (sMMO) and particulate MMO (pMMO). Notably, this oxidation process is unusual in requiring reducing equivalents: sMMO utilizes electrons from NADH (11–13), whereas pMMO is believed to accept electrons from reduced quinone species produced through NADH oxidation (14). Although methane serves as the primary substrate, some methanotrophs can also utilize methanol (14, 15). In contrast to methane utilization, direct utilization of methanol bypasses the electron-consuming methane oxidation step, potentially causing significant shifts in the intracellular redox balance (Fig. 1A). These redox perturbations suggest the involvement of redox-sensitive regulatory networks that adjust gene expression to facilitate metabolic reprogramming. However, in methanotrophs, the mechanisms underlying such regulatory systems remain largely uncharacterized.

The RegB/RegA (RegB/A) two-component system, consisting of the RegB histidine sensor kinase and the RegA global transcription regulator, is a highly conserved regulatory system in bacteria (16–18). Since its initial identification in *Rhodobacter capsulatus*, homologous systems have been identified in various species, including PrrB/A in *Cereibacter sphaeroides*, ActS/R in *Sinorhizobium meliloti*, and RoxS/R in *Pseudomonas aeruginosa* (16, 17). Acting as a redox sensor, the RegB/A system is influenced by oxygen availability, primarily activating its target genes under anaerobic or microaerobic conditions (17, 18). It regulates diverse cellular processes such as photosynthesis, CO₂ fixation, nitrogen assimilation, and the electron transport chain (17). In photosynthetic bacteria, such as *R. capsulatus* and *C. sphaeroides*, the RegB/A system is essential for the synthesis of photosystem under anaerobic conditions (16–18). Further research has also revealed the involvement of RegB/A in cytochrome biosynthesis, an essential component of the electron transport chain and respiration (17, 18). RegB/A also regulates genes involved in the oxidation of ferrous iron and inorganic sulfur compounds in *Acidithiobacillus ferrooxidans* (19). Beyond its metabolic functions, RegA plays a key role in bacterial pathogenicity, facilitating the adaptation of *Burkholderia pseudomallei* and *Brucella suis* to low-oxygen conditions within host environments (16, 20, 21). Furthermore, deletion of *regA* homolog in *P. aeruginosa* reduces its interaction with lung epithelial cells, suggesting a diminished pathogenic potential (22). Taken together, these findings highlight the central role of the RegB/A system in sensing oxygen availability and coordinating broad redox-regulated processes.

*Methylomonas* sp. DH-1, a type I methanotroph isolated from a brewery sludge plant, has gained attention for its fast growth, efficient methane-to-methanol conversion, and available genome annotation (23, 24). Genetic engineering of this strain has enabled the efficient conversion of methane into value-added products (3, 6, 10). In addition to methane, *Methylomonas* sp. DH-1 can utilize methanol as a sole carbon source, a physiologically distinct growth condition that is often associated with altered intracellular redox stress in methanotrophs. As methanol is the central intermediate in methane oxidation, understanding how methanotrophs adapt their metabolism and redox regulation during the transition from methane to methanol growth is of fundamental importance. Beyond its role as an intermediate of methane oxidation, methanol is increasingly recognized as an independent and industrially relevant C1 feedstock. Methanol can be readily produced from natural gas or CO_2_, is liquid at ambient conditions, and can be precisely supplied in bioprocesses, making it attractive for microbial bioconversion (25). Given that the RegB/A system functions as a key redox regulator in various bacteria, its role in methanotrophs may be central to understanding how these microbes regulate metabolism between methane and methanol. However, despite the presence of annotated RegB/A homologs in several methanotrophs, including *Methylomonas* sp. DH-1, their physiological roles and regulatory targets remain uncharacterized in any methanotrophic species to date.

In this study, we investigated the role of the RegB/A signaling system under methanol-grown conditions, providing the first evidence for a potential link between carbon source and redox control mediated by RegB/A in methanotrophs. Our findings demonstrate that iron uptake is essential for growth on methanol and that RegB/A activates iron uptake genes in response to methanol metabolism. Furthermore, we propose that this enhanced iron uptake supports the function of the Na^+^-transporting NADH:ubiquinone reductase, which helps alleviate excess NADH stress induced by direct methanol feeding.

## MATERIALS AND METHODS

### Strains and culture conditions

All strains used in this study are listed in Table S1. Strains were derived from *Methylomonas* sp. DH-1 (KCTC13004BP) and cultured in nitrate mineral salts (NMS) medium. The NMS medium consisted of 0.49 g/L MgSO_4_, 1.0 g/L KNO_3_, 0.23 g/L CaCl_2_·2H_2_O, 3.8 mg/L Fe-EDTA, 0.5 mg/L Na_2_MoO_4_, and 10 μM CuSO_4_·5H_2_O, with the addition of 1,000× trace element solution, 100× vitamin stock, and 100× phosphate stock solution. Detailed recipes of these solutions are provided in Table S2. Cultures were grown under three different conditions, all supplemented with 20% (vol/vol) methane of the headspace. Methane was supplied by gas injection using a gas-tight syringe, with injection volumes calculated based on the headspace volume of each culture vessel. The headspace volume was measured directly for each container, and the amount of methane supplied was calculated using the ideal gas law; for example, in a 125-mL flask containing 12.5 mL of culture, a 20% (vol/vol) methane in the headspace corresponds to approximately 1.24 mmol CH_4_. Specifically, cultures were grown: (i) in 3 mL of NMS medium in a 30 mL serum bottle sealed with a butyl rubber stopper (0.30 mmol CH_4_), (ii) in 12.5 mL of NMS medium in a 125 mL baffled flask sealed with a rubber-type screw cap (1.24 mmol CH_4_), or (iii) in 50 mL of NMS medium in a 500-mL baffled flask sealed with a rubber-type screw cap (4.96 mmol CH_4_). For methanol cultures, the same media volumes and flask sizes were used as in methane cultures, except 0.4% (vol/vol) methanol was supplied instead of methane (1.24 mmol CH_3_OH in 12.5 mL of NMS medium). The methanol concentration was calculated to provide a comparable molar amount of carbon to that supplied under methane-grown conditions. All cultures were incubated at 30°C with shaking at 170 rpm (3).

### Genetic manipulation of *Methylomonas* sp. DH-1

Plasmids and primers used in this study are listed in Tables S3 and S4. Plasmids for deleting genes in *Methylomonas* sp. DH-1 were generated based on the pIns plasmid, while plasmids for gene expression were generated based on the pFliE-mxaF plasmid (3, 26). Gene deletion plasmids, including pIns-regA_Del, pIns-tbdr_Del, pIns-tonB_Del, pIns-Na^+^-nqr_Del, and pIns-AYM39_16990_Del were constructed in two steps: (i) cloning a 1-kb upstream DNA fragment of the target gene (*regA, tbdr, tonB* operon, *Na^+^-nqr* operon, and *AYM39_16990*) into *Not*I*/Bcu*I or *Smi*I*/Bcu*I sites of pIns, followed by (ii) cloning a 1-kb downstream DNA fragment of each gene into *Apa*I*/Sac*I sites. DNA fragments used were prepared by PCR amplification from *Methylomonas* sp. DH-1 genomic DNA. The pIns-regB/A_Del plasmid was constructed using the same method as other deletion plasmids, with an additional step to exchange the selection marker from *kan^R^* to *amp^R^* by cloning using *Apa*I*/Pac*I.

pFliE-feoABC was constructed by cloning the *feo* operon into the *BamH*I*/Bcu*I sites of pFliE-mxaF, followed by marker change as in pIns-regB/A_Del. pFliE-T7-regA was generated by cloning the *regA* fragment with the T7 tag and G4S linker sequence into the *BamH*I*/Bcu*I sites of pFliE-mxaF; the T7 tag and G4S linker sequence were added before the *regA* sequence during PCR amplification via the forward primer. pFliE-regB/A was constructed by cloning [P*_regB/A_-regB-regA*] cassette from DH-1 genomic DNA into the *Sgs*I*/Bcu*I sites of pFliE-mxaF. The plasmids pFliE-regB^∆UBD^/A, pFliE-regB^H239A^/A, and pFliE-regB/A^D59A^ were generated from pFliE-regB/A using PCR-based mutagenesis. Gene deletion or insertion in *Methylomonas* sp. DH-1 was performed as previously described via homologous recombination into the chromosome (3).

### Quantitative reverse transcription PCR and RNA-seq

Total RNA of *Methylomonas* sp. DH-1 and Δ*regA* strain was extracted with minor modifications to previously described methods (3). Strains were cultured in 12.5 mL of NMS medium supplemented with methane or methanol, and samples were collected at the early exponential phase when OD_600_ reached 1.0–1.5. RNA libraries were constructed using the Illumina TruSeq Stranded RNA Library Construction kit and sequenced on an Illumina NovaSeq 6000 S4 platform. For RNA-seq data analysis, genes with a count of zero in any of the samples were filtered out to ensure statistical robustness. The remaining raw counts were normalized using Trimmed Mean of M-values (TMM) method via the calcNormFactors function in the edgeR R library to minimize systematic biases. Fold change (Fc) values were calculated through pairwise comparisons between specific experimental conditions to identify differentially expressed genes: (i) methanol-grown versus methane-grown wild-type cells to identify carbon source-dependent changes and (ii) methanol-grown Δ*regA* versus methanol-grown wild-type cells to identify RegA-dependent regulation. Quantitative reverse transcription-PCR (qRT-PCR) analysis and RNA-Seq were performed as previously described, using the *glgA* gene (*AYM39_03770*) for normalization in qRT-PCR analysis, which showed stable expression under the experimental conditions (26). Primers for qRT-PCR are listed in Table S3.

### Quantification of cellular iron concentrations

Intracellular iron content of *Methylomonas* sp. DH-1 was quantified by inductively coupled plasma mass spectrometry (ICP-MS) and normalized to dry cell weight. Cells were harvested when OD_600_ reached 1.0–1.5, then washed three times with PBS (pH 7.4; Welgene, Republic of Korea). Dried cell pellets were digested according to the EPA3051A method prior to ICP-MS analysis.

### Chromatin immunoprecipitation

Chromatin immunoprecipitation (ChIP) was conducted as previously described with minor modifications using *Methylomonas* sp. DH-1 RegA^T7-tagged^ (26). When OD_600_ reached 1.0–1.5, corresponding to the same growth phase used for qRT-PCR and RNA-Seq analyses, formaldehyde (2.7%) was added to 50 mL of culture using a syringe and crosslinked for 25 min, followed by quenching with 250 mM glycine for 5 min. Cells were washed once with ice-cold NMS, three times with TBS, and once with lysozyme buffer (10 mM Tris-HCl [pH 8.0], 20% sucrose, 50 mM NaCl, 10 mM EDTA), then resuspended in 500 μL of lysozyme buffer containing 10 mg/mL of lysozyme (Thermo Scientific, USA), 1 mM PMSF, and 0.1% protease inhibitor cocktail. After lysis at 37°C for 30 min, 500 μL of 2× ChIP lysis buffer (100 mM HEPES-KOH [pH 7.5], 300 mM NaCl, 2 mM EDTA, 2% Triton X-100, 0.2% sodium deoxycholate, 0.4% SDS) was added, and the lysate was sonicated 15 times for 20 s (Vitra-cell, Sonics & Materials Inc., USA) at 22% amplitude. Crude lysates were centrifuged for 20 min, and 150 μL of supernatant was used as input. For immunoprecipitation, 2 μL of anti-T7 tag antibody was added to 600 μL of lysate and incubated overnight at 4 °C, followed by a 2-h incubation with 20 μL Protein A Plus agarose bead (Santa Cruz Biotechnology, USA). After washing the beads, DNA was eluted from the beads and treated with RNase and proteinase K. Crosslink was reversed by overnight incubation at 65°C with 100 mM NaCl, and DNA was purified using the Qiagen DNA purification kit. RegA occupancy at target promoters was quantified by comparing the relative enrichment of immunoprecipitated DNA to input DNA and was normalized to a negative control locus (*glgA* ORF) to assess background binding. The primers used in ChIP-qPCR are listed in Table S3.

### NAD^+^/NADH ratio calculation

The intracellular concentrations of NAD^+^ and NADH were quantified using the PicoSens NAD^+^/NADH Assay Kit (Biomax), following the manufacturer’s instructions. Harvested cells were disrupted by two cycles of freeze-thawing, and cell debris was removed by centrifugation at 4°C. The resulting supernatant was divided into two aliquots: one was used to measure the total amount of NAD^+^ and NADH, and the other was incubated at 60°C for 30 min to degrade NAD^+^, allowing for the specific measurement of NADH. Both aliquots were then mixed with cycling enzymes that convert NAD^+^ to NADH, followed by the addition of a colorimetric probe. After 1 h of incubation, absorbance was measured at 450 nm (OD_450_), and the NAD^+^/NADH ratio was calculated accordingly.

## RESULTS

### RegB/A system regulates methanol metabolism in *Methylomonas* sp. DH-1

While *Methylomonas* sp. DH-1 can utilize both methane and methanol as carbon sources, these substrates impose distinct intracellular redox states. This strain has only pMMO and lacks sMMO for methane oxidation. Methane oxidation via pMMO requires input of reducing equivalents (electrons) to activate molecular oxygen for converting methane to methanol (Fig. 1A). In contrast, direct utilization of methanol as a carbon source bypasses the electron-consuming methane oxidation step catalyzed by MMO (Fig. 1A), which may lead to redox imbalance during growth on methanol. Although the RegB/A system is traditionally associated with oxygen sensing in other bacteria, the redox imbalance, caused by the accumulation of excess reducing equivalents during methanol metabolism, may serve as a physiological cue for RegB/A activation.

To investigate the role of the RegB/A system in *Methylomonas* sp. DH-1, we first examined the conservation of key functional features of RegB and RegA based on the established RegB/A regulatory model (Fig. 1B) (17). Sequence homology analysis revealed that essential functional domains, including the ubiquinone-binding domain of RegB (GGASNPI), the autophosphorylation site of RegB (His239), and the phosphorylation site of RegA (Asp59), are conserved in *Methylomonas* sp. DH-1 (Fig. 1C) (17, 27).

**Fig 2:**
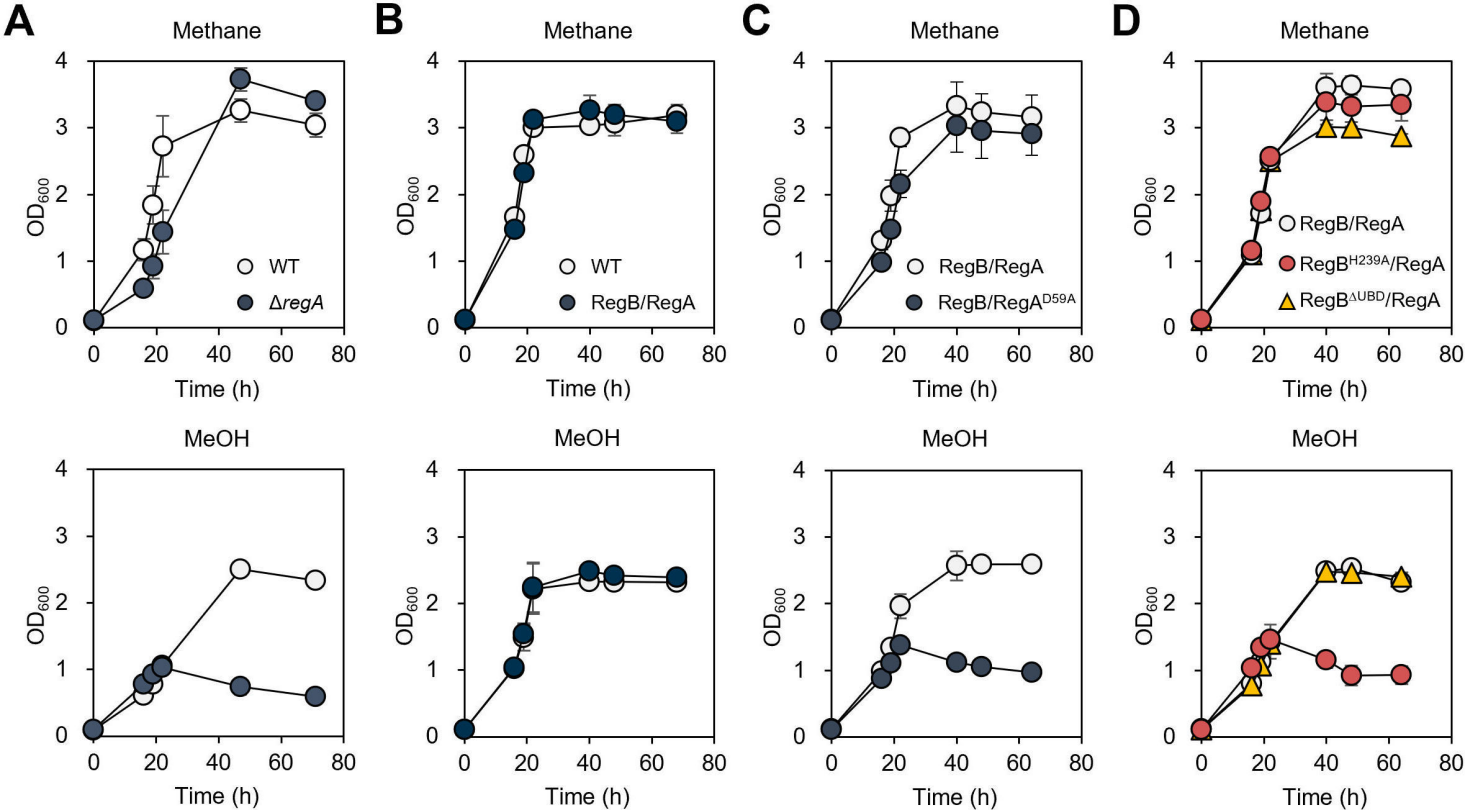
Role of the RegB/A signaling pathway during methanol growth. (**A–D**) Growth curves of the indicated strains cultured in NMS media with different carbon sources. 20% (vol/vol) methane was used for methane feeding, and 0.4% methanol was used for methanol feeding. Error bars represent standard deviation (s.d.) from three biological replicates (*n* = 3).

To explore the potential role of the redox-responsive RegB/A system under methane and methanol growth conditions, we deleted *regA*, which encodes the response regulatory transcription factor of the RegB/A system. The growth phenotype of the resulting Δ*regA* mutant was compared with that of the wild type in NMS medium supplemented with either 20% (vol/vol) methane or 0.4% methanol, providing an equimolar amount of carbon in both conditions. Compared with growth on methane, the wild-type strain exhibited a lower growth rate and reduced final biomass when grown on methanol (Fig. 2A). Under methane-grown conditions, the Δ*regA* mutant showed a modest decrease in exponential-phase growth but ultimately reached biomass levels similar to the wild type. In contrast, the Δ*regA* strain exhibited a severe growth defect when cultured with methanol as the carbon source, suggesting the essential role of the RegB/A system in methanol metabolism (Fig. 2A). Given that RegA functions as a global transcriptional regulator, the growth defect observed in the Δ*regA* strain is likely caused by the loss of gene regulation that directly or indirectly mitigates redox imbalances during methanol metabolism.

To further elucidate the activation mechanisms of the RegB/A system in methanol metabolism, we examined the effects of disrupting specific steps in its signal transduction cascade, as illustrated in Fig. 1B. In the canonical RegB/A regulatory model, signal transduction is mediated through autophosphorylation of RegB followed by phosphotransfer to RegA, while the activity of RegB is modulated by quinone/quinol binding via the ubiquinone-binding domain (UBD). Based on this model, we targeted three key steps in the RegB/A pathway-quinone/quinol binding, RegB autophosphorylation, and RegA phosphorylation-to dissect their roles during methanol growth. To this end, we deleted the entire *regB/A* operon and then reintroduced modified versions of the *regB/A* operon into the *fliE* locus, each harboring a mutation that disrupts a specific regulatory function: RegB^ΔUBD^, which blocks ubiquinone binding to RegB; RegB^H239A^, which prevents autophosphorylation of RegB; and RegA^D59A^, which abolishes phosphorylation of RegA. As a control, the wild-type *regB/A* operon was also integrated into the *fliE* locus of the Δ*regB/A* strain, generating the RegB/RegA strain. The wild-type and RegB/RegA strains exhibited comparable growth under both methane and methanol conditions (Fig. 2B).

We next examined the growth phenotypes of the engineered strains in NMS medium supplemented with either methane or methanol. Strains harboring mutations that disrupted phosphotransfer-RegB^H239A^/RegA and RegB/RegA^D59A^-exhibited growth defects under methanol conditions, closely resembling the phenotype of the Δ*regA* strain (Fig. 2C and D). This indicates that the phosphorelay activity of both RegB and RegA is essential for the proper function of the RegB/A system. In contrast, disruption of ubiquinone/ubiquinol binding in the RegB in RegB^ΔUBD^/RegA strain did not impair methanol growth (Fig. 2D). This absence of a growth defect indicates that ubiquinol (QH_2_, reduced state) binding is not required for RegB activation under methanol-grown conditions. Instead, these results support a model in which RegB retains basal kinase activity in the absence of inhibitory ubiquinone (Q, oxidized state) binding, consistent with previous observations in *R. capsulatus* (27). In this context, RegB remains competent for autophosphorylation and subsequent phosphotransfer to RegA when ubiquinone-mediated repression is relieved. In contrast to the pronounced effects observed under methanol conditions, the RegB^H239A^/RegA and RegB/RegA^D59A^ mutant strains exhibited growth patterns comparable to the RegB/RegA strain under methane culture conditions (Fig. 2C and D). However, the RegB^ΔUBD^/RegA strain showed reduced biomass accumulation during the stationary phase.

Taken together, our results suggest that the RegB/A system senses redox imbalance under methanol conditions and transduces this signal through its conserved phosphorelay pathway to regulate methanol metabolism.

### RegA upregulates the genes responsible for iron uptake in methanol culture

**Fig 3:**
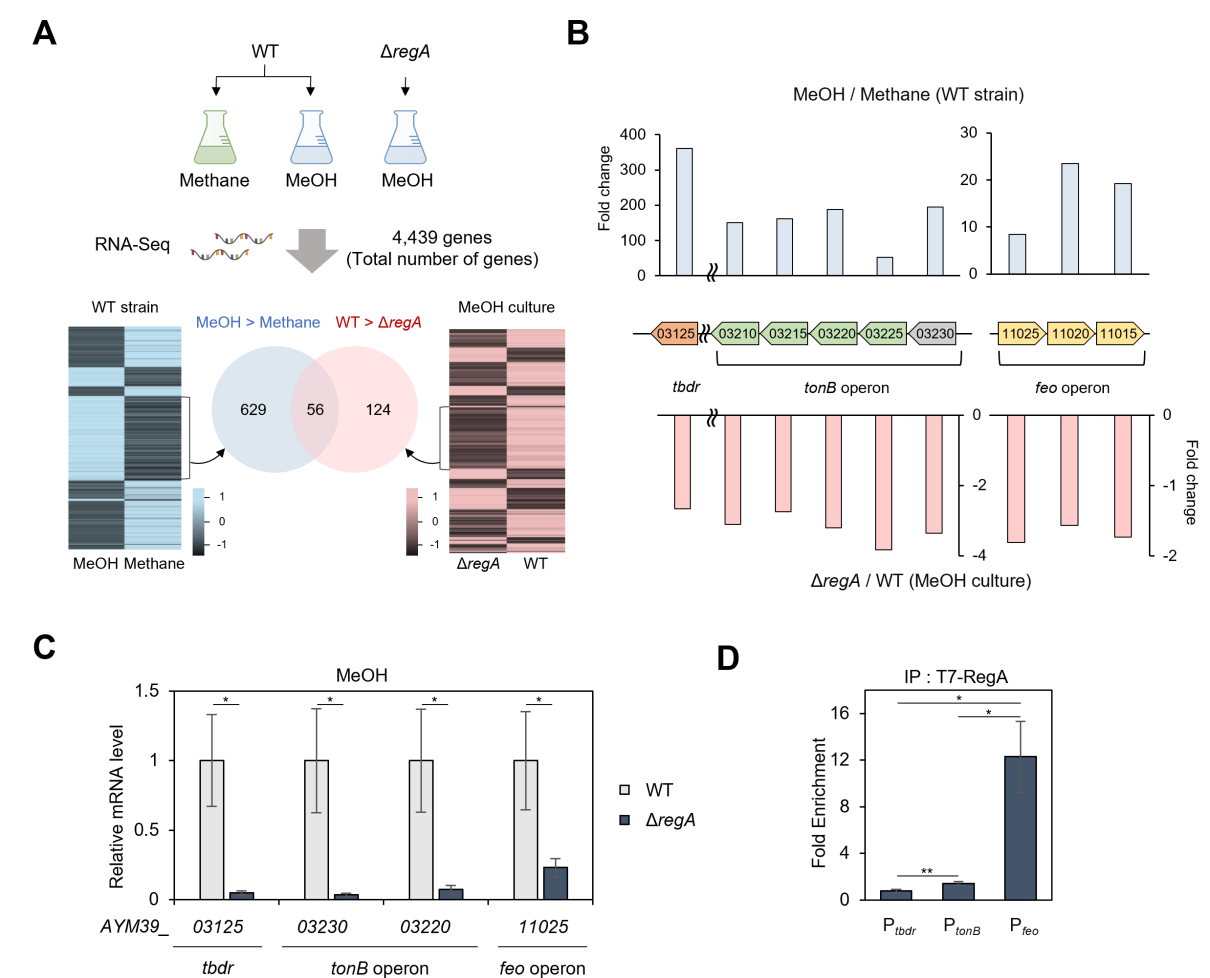
RegA-mediated upregulation of iron uptake genes during methanol growth. (**A**) Schematic overview of the RegA target selection process based on RNA-Seq analysis. A total of 4,439 genes were included in the entire transcriptomic analysis. (**B**) Putative RegA targets identified using two criteria: (i) genes upregulated during methanol growth, and (ii) genes with lower expression in the Δ*regA* strain than in the wild type under methanol culture conditions. (**C**) Relative expression levels of target genes in the indicated strains under methanol culture conditions. Error bars represent s.d. (*n* = 3). Statistical significance was determined by a two-tailed Student’s t-test (*P < 0.05) (**D**) ChIP analysis of T7-RegA binding to target promoters. Each interaction was normalized to that of the T7-RegA with the negative control gene (*glgA* ORF). Error bars represent s.d. (*n* = 3). Statistical significance was determined by a two-tailed Student’s t-test (*P < 0.05; **P < 0.01).

To understand the role of the RegB/A system in methanol metabolism, we conducted RNA-seq analysis to identify genes regulated by RegA (Table S5). Wild-type cells were cultivated with either methane or methanol to determine carbon source-dependent gene expression profiles. Additionally, the Δ*regA* strain was grown with methanol to identify RegA-dependent transcriptional changes under methanol-utilizing conditions (Fig. 3A). To narrow down potential RegA target genes, we established two criteria: (i) genes upregulated in wild type during methanol growth and (ii) genes with reduced expression in the Δ*regA* strain than in the wild type during methanol growth (Fig. 3A). For the first criterion, we used a fold change (Fc) >2 and a *P*-value < 0.05; for the second criterion, a more relaxed threshold of Fc >1.5 and a *P*-value < 0.05 was applied. While we initially intended to use identical thresholds, the transcriptional impact of *regA* deletion was less pronounced compared to the transcriptional changes induced by the carbon source variation, prompting the use of a lower cutoff for the second criterion. Out of 4,439 genes analyzed, 685 met the first criterion, 180 met the second, and only 56 satisfied both, identifying them as strong candidates for direct or indirect regulation by RegA (Fig. 3A; Table S6). After excluding genes encoding proteins of unknown function from the 56 candidates, the remaining genes could be grouped into a few operons primarily responsible for iron uptake.

As shown in Fig. 3B, three genomic regions satisfied both criteria and exhibited significant differential expression: (i) the TonB-dependent siderophore receptor (TBDR, *AYM39_03125*); (ii) the *tonB* operon (*AYM39_03230 - AYM39_03210*), which consists of periplasmic protein TonB and biopolymer transporter proteins (ExbB, ExbD); and (iii) the *feo* operon (*AYM39_11025 - AYM39_11015*). The *tbdr* and *tonB* operons are associated with Fe^3+^ uptake, while the Feo system mediates Fe^2+^ transport. In gram-negative bacteria, iron is typically acquired in its ferric form (Fe^3+^) via siderophore-mediated uptake through TonB-dependent receptors in the outer membrane, after which it is reduced to ferrous iron (Fe^2+^) for cellular use (28, 29). Additionally, Fe^3+^ can be reduced to Fe^2+^ in the periplasm or even in the extracellular space by ferric reductases, after which Fe^2+^ is transported into the cytoplasm via the Feo system (30). Consistent with the transcriptional upregulation of these iron uptake systems, ICP-MS analysis revealed that cells grown under methanol conditions contained a higher intracellular iron content than those grown on methane when normalized to dry cell weight (Fig. S1). Together, these results indicate that methanol growth is associated with increased cellular iron demand, as reflected both at the transcriptional level and by elevated intracellular iron accumulation.

We validated RegA-dependent regulation of these target genes using qRT-PCR. In methanol-grown cells, the expression levels of *tbdr* (*AYM39_03125*), the *tonB* operon (*AYM39_03230, AYM39_03220*), and the *feo* operon (*AYM39_11025*) were reduced to 3%–23% of wild-type levels in the Δ*regA* strain (Fig. 3C). Expression of *glgA*, which remained stable between the wild-type and *ΔregA* strains under these conditions, was used as an internal normalization control. Next, we determined whether RegA directly regulates these target genes. We introduced a T7 tag into the genomic copy of *regA* and conducted chromatin immunoprecipitation (ChIP) under methanol growth conditions. This analysis revealed a significant enrichment of the *feo* operon promoter, while the promoters of the *tbdr* gene and the *tonB* operon showed no appreciable binding by RegA (Fig. 3D). These findings suggest that RegA directly activates the *feo* operon during methanol metabolism, whereas its effects on other target genes may be indirect.

### Iron uptake mediated by the Feo system is essential for methanol metabolism

**Fig 4:**
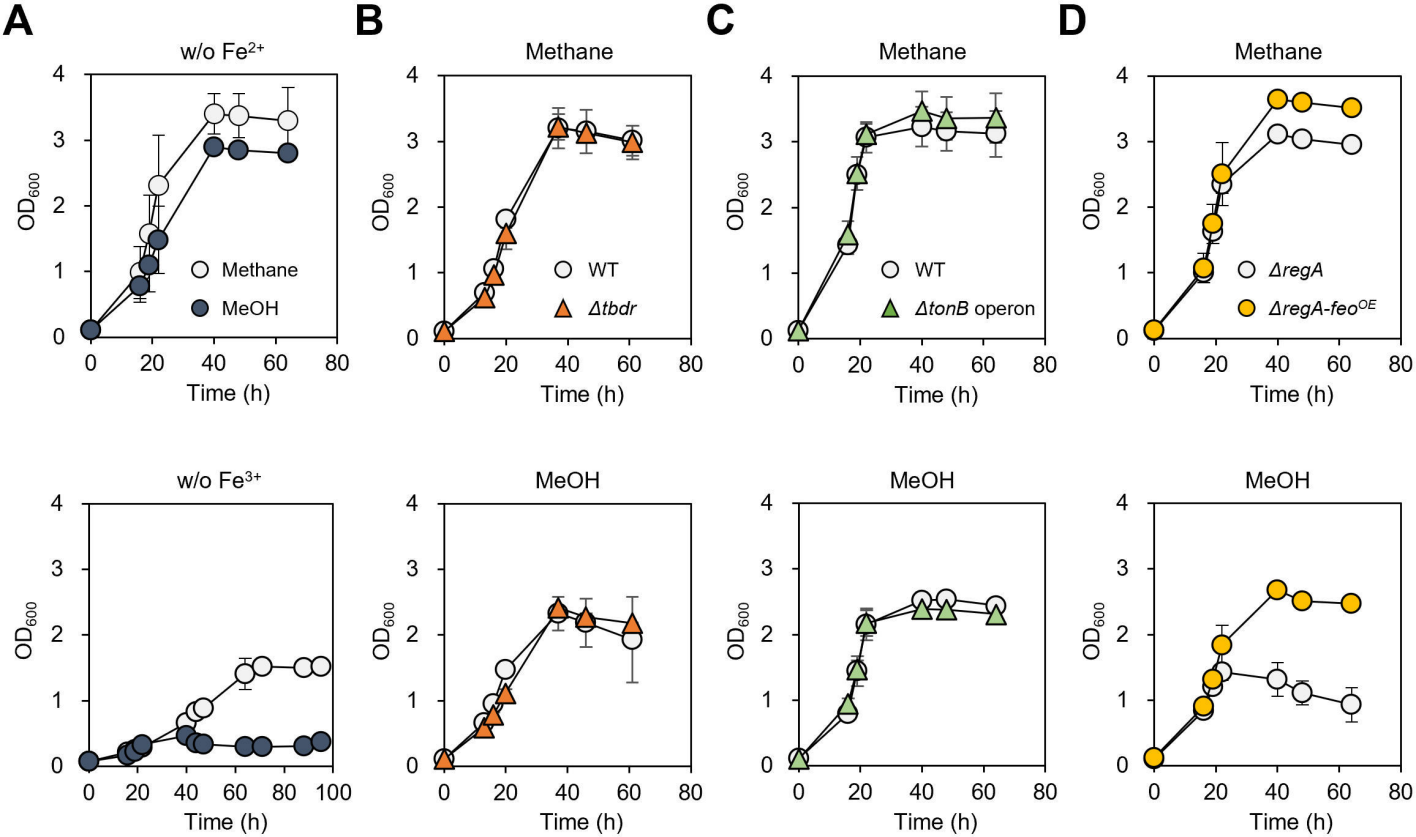
Requirement of iron uptake for methanol growth. (**A**) Growth curve of wild-type strain in NMS medium lacking either Fe^3+^ or Fe^2+^, with different carbon sources. Error bars represent s.d. (*n* = 3). (**B–D**) Growth curves of the indicated strains in NMS media with different carbon sources. Error bars represent s.d. (*n* = 3).

Given that the identified RegA target genes are involved in iron uptake, we examined the roles of Fe^3+^ and Fe^2+^ ions in methanol metabolism. To this end, the wild-type strain was cultured in iron-depleted NMS medium under both methane and methanol growth conditions. The standard NMS medium contains two iron sources: Fe-EDTA, the primary source supplying 10.35 µM Fe^3+^, and FeSO_4_•7H_2_O, a secondary source providing 1.80 µM Fe^2+^. The omission of FeSO_4_•7H_2_O (Fe^2+^) had no noticeable effect on growth under either methane or methanol conditions (Fig. 4A) compared with growth in NMS medium (Fig. 2A), indicating that exogenous Fe^2+^ plays a negligible role. In contrast, the absence of Fe-EDTA (Fe^3+^) hindered cellular growth under both methane and methanol conditions (Fig. 4A); however, the inhibitory effect was more pronounced in methanol-grown cells, underscoring the importance of Fe^3+^ uptake during methanol metabolism (Fig. 4A). These results indicate that the primary iron source Fe^3+^ may be efficiently reduced to Fe^2+^ either extracellularly or within the periplasm, ensuring sufficient iron availability for cellular functions.

Next, we evaluated the roles of the predicted RegA target genes involved in iron uptake in methanol metabolism. Deletion of *tbdr* and *tonB* operon, which are supposed to be indirectly regulated by RegA, did not affect cell growth under either methane or methanol conditions compared to wild type (Fig. 4B and C). Nevertheless, this observation does not rule out the possibility that these genes are required for methanol metabolism, as multiple *tonB* and *tbdr* homologs are present in the *Methylomonas* sp. DH-1 genome may compensate for the gene deletions. The *feo* operon was identified as a direct target of RegA. To investigate its role in RegA-mediated regulation, we attempted to delete the operon; however, repeated efforts were unsuccessful. Given that gene deletion was performed under methane-grown conditions, it suggests that the *feo* operon is essential for general cellular viability regardless of the carbon source. This essentiality is likely due to the fundamental requirement of iron for various core metabolic enzymes, even during growth on methane. If the *feo* operon functions as a downstream effector of RegA in methanol metabolism, its overexpression might compensate for the growth defect observed in the Δ*regA* strain. To test this hypothesis, we overexpressed the *feo* operon in the Δ*regA* background under the control of the strong *mxaF* promoter, generating the Δ*regA-feo^OE^* strain. Notably, the methanol-specific growth defect of the Δ*regA* mutant was rescued in the Δ*regA-feo^OE^* strain, supporting the conclusion that the *feo* operon acts as a downstream target of RegA regulation and is essential for growth on methanol (Fig. 4D). Although the supplementation of Fe^3+^, but not Fe^2+^, was required to support methanol-dependent growth (Fig. 4A), our findings indicate that the Feo system plays a critical role in supplying intracellular Fe^2+^ necessary for key biological processes.

### Na^+^-transporting NADH:ubiquinone reductase containing iron–sulfur cluster is essential for methanol metabolism

Given the observed requirement of iron uptake under methanol conditions, we next investigated the underlying basis for this increased demand. We hypothesized that iron-containing proteins may play a critical role in methanol metabolism. Among the 56 candidate RegA target genes we identified (Fig. 3A), one gene encoded a putative 2Fe-2S-binding protein (*AYM39_16990*) (Fig. S2A). However, deletion of this gene did not affect growth under methanol conditions (Fig. S2B), suggesting that it does not play a major role in methanol metabolism.

**Fig 5:**
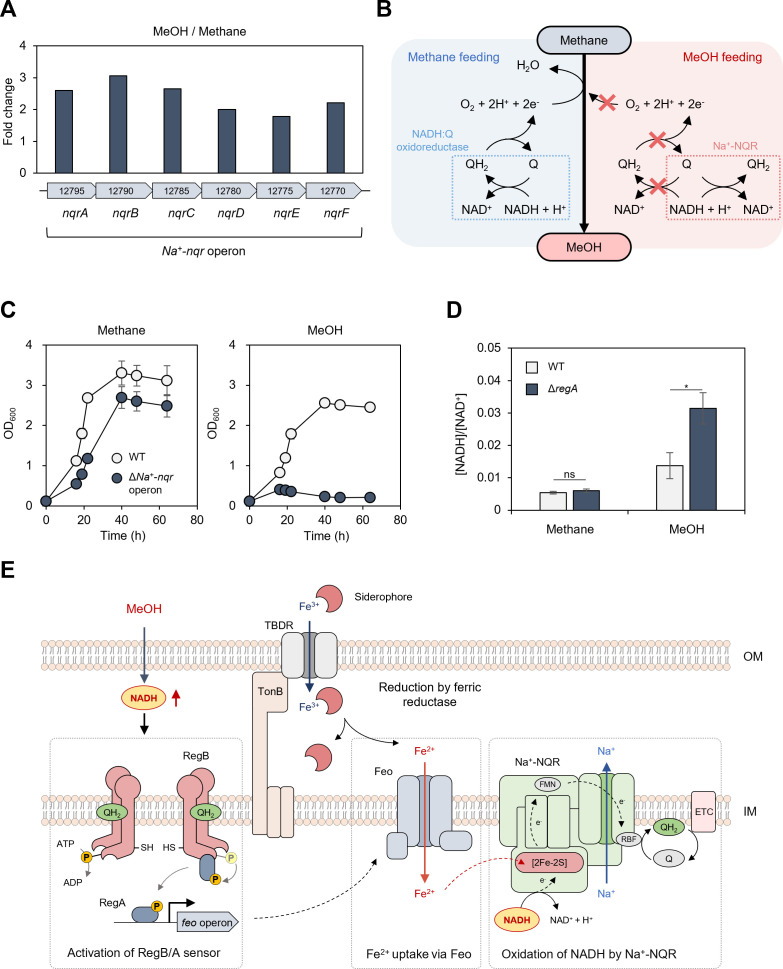
Na^+^-NQR alleviates excess NADH stress during methanol growth. (**A**) Expression levels of the *Na^+^-nqr* operon under methanol culture conditions relative to methane. (**B**) Schematic comparison of methane and methanol metabolism. (**C**) Growth curves of the indicated strain cultured in NMS media with different carbon sources. Error bars represent s.d. (*n* = 3). (**D**) Intracellular [NADH]/[NAD^+^] ratios in the indicated strains under different carbon sources. Error bars represent s.d. (*n* = 3). Statistical significance was determined by a two-tailed Student’s t-test (*P < 0.05; ns, not significant). (**E**) Proposed mechanism by which Na^+^-NQR alleviates excess NADH stress under methanol conditions.

To further investigate the connection between iron uptake and methanol metabolism, we analyzed all 685 methanol-induced genes in *Methylomonas* sp. DH-1 and, based on manual curation of KEGG pathway annotations and predicted protein functions, identified 99 proteins that either require iron as a cofactor or interact with it. Among these, we screened for genes related to ubiquinone or NADH metabolism, as these cofactors are likely affected by redox balance shifts during methane and methanol utilization, and identified a single operon encoding the Na^+^-transporting NADH:ubiquinone reductase (Na^+^-NQR) complex, hereafter referred to as the *Na^+^-nqr* operon (Fig. 5A). This operon was upregulated under methanol conditions in both the wild-type and Δ*regA* strains. Na^+^-NQR, composed of six subunits, transfers electrons from NADH to quinone while exporting Na^+^ to the periplasm, thereby contributing to NADH oxidation. Notably, this process involves an iron–sulfur cluster that functions as an electron carrier (31–33).

Based on these findings, we hypothesized that Na^+^-NQR is upregulated under methanol conditions to mitigate NADH accumulation and that this upregulation increases cellular iron demand, which is met through RegB/A-mediated upregulation of iron uptake systems (Fig. 3B, C and 5B). To test this hypothesis, we deleted the *Na^+^-nqr* operon and assessed its effect on methane or methanol culture. While the mutant exhibited only mild growth inhibition in methane conditions, it failed to grow under methanol conditions, resembling the severe growth defect observed in the Δ*regA* strain (Fig. 2A and 5C).

The absence of Na^+^-NQR may impair the cell’s ability to efficiently consume excess NADH during methanol metabolism, ultimately resulting in cell death. If NADH accumulation is the primary cause of methanol toxicity, then even low concentrations of methanol, without co-supplied methane, should severely affect strains lacking NADH detoxification mechanisms. To test whether excess NADH impairs growth even at low methanol levels, we compared wild-type and Δ*Na^+^-nqr* strains across a range of concentrations. The wild type showed reduced but measurable growth at 0.1% methanol-likely limited by carbon availability, whereas the Δ*Na^+^-nqr* strain failed to grow at any concentration (Fig. S3), indicating that impaired NADH oxidation compromises viability even under minimal methanol exposure.

To validate the proposed link between RegA and NADH redox imbalance, we measured the intracellular NADH/NAD^+^ ratio in the wild type and the Δ*regA* strain under both methane and methanol culture conditions (Fig. 5D). Under methane culture, the NADH/NAD^+^ ratios were comparable between the two strains. In contrast, methanol culture led to increased NADH/NAD^+^ ratios in both strains, indicating that methanol metabolism intrinsically promotes NADH accumulation. Notably, this increase was much more pronounced in the Δ*regA* strain, indicating that RegA is a key regulator essential for managing methanol-induced redox stress. Collectively, these results demonstrate the essential function of RegA in maintaining redox homeostasis by mitigating NADH-induced stress under methanol growth conditions via enhancing Feo-dependent uptake of Fe^2+^, required for the activity of iron–sulfur containing Na^+^-NQR (Fig. 5E).

## DISCUSSION

Maintaining redox balance, often reflected by the intracellular NADH/NAD^+^ ratio, is a fundamental requirement for cellular metabolism across all domains of life, from microbes to humans (34, 35). While NADH serves as a key electron donor in energy production via the electron transport chain (ETC), its excessive accumulation is toxic to the cells. Elevated NADH levels inhibit the TCA cycle, thereby disrupting not only energy metabolism but also essential biosynthetic processes such as amino acid (e.g., aspartate) and protein synthesis, ultimately leading to cellular toxicity and growth defects (36). Importantly, previous studies have shown that restoring the NADH/NAD^+^ ratio by oxidizing excess NADH without generating ATP can rescue cell growth (36, 37).

This redox balance is particularly critical in methanotrophs, as NADH serves either directly or indirectly as an electron donor for methane oxidation via MMO. In *Methylomonas* sp. DH-1, methanol metabolism presents a distinct redox challenge. Unlike methane oxidation, which consumes electrons and helps maintain intracellular redox homeostasis, methanol metabolism lacks this electron sink, leading to the accumulation of reduced cofactors such as NADH. In *Methylococcus capsulatus* (Bath), NADH oxidation to NAD^+^ has been observed to occur in coupling with the methane oxidation, understanding its role as a redox buffer (38).

Our study reveals that the RegB/A two-component system plays a central role in redox sensing and adaptation during methanol assimilation in *Methylomonas* sp. DH-1. RegB/A, initially characterized as an oxygen-responsive system in photosynthetic bacteria, is now recognized as a broader redox regulator (17–19, 39–41). In *Methylomonas* sp. DH-1, a strict aerobe, RegB/A is activated not by oxygen limitation but by the carbon source, specifically under methanol-induced redox stress, likely through elevated NADH level, which is likely accompanied by an increase in ubiquinol (QH_2_) levels.

Transcriptomic analysis identified the *feo* operon, encoding a Fe^2+^ transporter, as a RegA target essential for methanol metabolism. Increased iron uptake facilitated by the Feo transporter enhances the activity of the Na^+^-NQR complex, a NADH-oxidizing enzyme containing iron–sulfur clusters. Increased Fe^2+^ availability supports Na^+^-NQR function, enabling efficient NADH oxidation and thereby alleviating redox stress during methanol growth. While previous reports have linked RegA to iron uptake regulation in *R. capsulatus* and iron oxidation pathways in *A. ferrooxidans*, our findings reveal a distinct regulatory mechanism in *Methylomonas* sp. DH-1 (19, 41). Notably, although our results indicate that RegA directly binds only to the promoter of the *feo* operon, the observed downregulation of *tbdr* and *tonB* operons in the Δ*regA* strain suggests that these genes are also under the control of the RegB/A system, likely via an indirect regulatory mechanism. The specific intermediary factors or signaling pathways responsible for this indirect regulation remain to be identified and warrant future investigation.

Interestingly, although Fe^2+^ uptake is upregulated via Feo, methanol-specific growth defects occur under Fe^3+^-limiting conditions. As iron uptake mechanisms in methanotrophs are poorly characterized, we referred to the well-established systems in gram-negative bacteria such as *E. coli* (29). In these organisms, Fe^3+^, which is less soluble than Fe^2+^, is typically acquired via siderophores. The Fe^3+^-siderophore complex is transported into the periplasm by TonB-dependent outer membrane receptors, then imported into the cytoplasm via ATP-binding cassette (ABC) transporters in the inner membrane (42–44). Ferric reductases can also liberate iron from the Fe^3+^-siderophore complex and reduce it to Fe^2+^, which is subsequently transported into the cytoplasm by the Feo system (30, 44, 45). Given the essential roles of the Feo system and Fe^3+^ supplementation in methanol growth, Feo-dependent Fe^2+^ uptake is likely the primary iron acquisition route in *Methylomonas* sp. DH-1.

Another critical aspect of the redox physiology of *Methylomonas* sp. DH-1 is the presence of only pMMO, but not sMMO. A fundamental distinction between sMMO and pMMO lies in their cofactor requirements and integration into the cellular redox network. sMMO is an iron-containing, cytoplasmic enzyme that directly utilizes NADH and contains redox-active cofactors such as FAD and iron–sulfur clusters (46). In contrast, pMMO is a copper-dependent, membrane-bound complex that lacks intrinsic redox cofactors and does not interact directly with NADH. Instead, it receives electrons indirectly via the NADH:quinone oxidoreductase system (8, 47). This pathway allows NADH oxidation to be coupled with energy conservation via the generation of a proton motive force before electrons reach the pMMO active site (48). Additionally, pMMO has a lower *K_m_* for methane than sMMO (1, 8). Due to this efficiency, methanotrophs preferentially express pMMO when copper is available. In *M. capsulatus* (Bath), the transition from sMMO to pMMO expression has been shown to improve growth by reducing the direct demand for NADH during methane oxidation (48). However, this reliance on pMMO may also increase susceptibility to NADH accumulation, particularly under conditions where NADH is produced in excess and reoxidation capacity is limited. This vulnerability is likely exacerbated in *Methylomonas* sp. DH-1, which lacks sMMO and therefore cannot alleviate redox stress through direct NADH-dependent methane oxidation (15).

Consequently, cells expressing pMMO must rely on auxiliary systems to re-oxidize NADH and maintain redox balance during methane or methanol metabolism. One such auxiliary system is the Na^+^-NQR, which simultaneously oxidizes NADH and exports Na^+^ across the membrane, generating a sodium motive force (SMF). This system is particularly prevalent in bacteria inhabiting marine and alkaline environments, where sodium gradients are favored over proton gradients for energy conservation (49, 50). In *Methylomonas* sp. DH-1, our findings indicate that Na^+^-NQR plays a critical role in alleviating NADH accumulation under methanol growth conditions. Because Na^+^-NQR directly oxidizes NADH and reduces quinone, this mechanism provides a means to alleviate redox imbalance independently of increased terminal respiratory activity. This contrasts with the canonical redox balancing via oxidative phosphorylation and proton-motive force (PMF), which depends on electron flux through downstream pathways such as the TCA cycle. Because redox stress can arise rapidly upon carbon source uptake, particularly in methanol metabolism lacking intrinsic electron sinks, cells require an early-acting mechanism to promptly regenerate NAD^+^ and sustain metabolic activity. Na^+^-NQR may fulfill this role by providing immediate capacity for NADH oxidation, independent of slower downstream processes.

The distribution of Na^+^-NQR across methanotrophs supports the potential role of SMF in maintaining redox balance. For instance, *Methylocella* species, which possess only sMMO, lack annotated homologs of Na^+^-NQR, suggesting that redox homeostasis in these organisms can be achieved through the intrinsic activity of sMMO (51). In contrast, Na^+^-NQR homologs are consistently found in methanotrophs harboring pMMO (52, 53). Although the mechanistic link between SMF and pMMO remains unclear, genomic and expression patterns imply a functional association, indicating that Na^+^-NQR may help mitigate redox stress in pMMO-dominant systems.

Overall, our results suggest a coordinated regulatory strategy in which *Methylomonas* sp. DH-1 manages methanol-induced redox stress. Growth on methanol leads to the accumulation of NADH, prompting the induction of the Na^+^-NQR complex as a primary redox-buffering mechanism to alleviate excess NADH stress. Because Na^+^-NQR activity depends on iron–sulfur clusters, this metabolic shift inherently increases the cellular demand for iron. The RegB sensor kinase recognizes the elevated reduction state of the quinone pool (QH_2_) resulting from this buffering process and activates RegA, which subsequently upregulates the *feo* operon to enhance iron uptake. In this way, the RegB/A system ensures that sufficient iron is available to support Na^+^-NQR activity. To maintain this redox buffering cycle, the accumulated QH_2_ may subsequently be oxidized through the electron transport chain, ultimately transferring electrons to molecular oxygen via terminal respiratory oxidases and thereby restoring the ubiquinone pool. This integrated regulatory response coordinates RegA-independent methanol-induced expression of the Na^+^-NQR machinery with RegA-dependent iron acquisition, ultimately restoring redox homeostasis during methanol metabolism. Methanotrophs hold significant biotechnological potential due to their dual ability to reduce greenhouse gas emissions and convert inexpensive, abundant C1 substrates into valuable chemicals. However, industrial applications have been constrained by limited insights into their fundamental physiology. This study provides a comprehensive characterization of the RegB/A two-component system and its role in coordinating redox-responsive regulation during growth on methanol, a physiologically distinct condition in *Methylomonas* DH-1. By uncovering how this regulatory system links environmental cues to intracellular redox balance, our findings advance the foundational understanding of methanotroph biology, providing a critical framework for engineering robust, redox-adaptive methanotrophic platforms for sustainable bioproduction.

## Data Availability

All data generated or analyzed during this study are included in this published article and its supplemental material.

## SUPPLEMENTAL MATERIAL

Supplemental material aem.00495-26-s0001.docx Tables S1 to S4; Fig. S1 to S3.

Table S5 aem.00495-26-s0002.xlsx TMM normalized transcriptome profiling.

Table S6 aem.00495-26-s0003.xlsx RNA-Seq (final candidates).

ASM does not own the copyrights to Supplemental Material that may be linked to, or accessed through, an article. The authors have granted ASM a non-exclusive, world-wide license to publish the Supplemental Material files. Please contact the corresponding author directly for reuse.
