# Supplementary material for: RegB/A-dependent redox regulation links iron uptake to methanol metabolism in Methylomonas sp. DH-1
Source: Appl Environ Microbiol. 2026 May 19;92(6):e00495-26. doi: 10.1128/aem.00495-26 (PMC13274454; doi:10.1128/aem.00495-26)
Supplement: Supplemental material — Tables S1 to S4; Fig. S1 to S3. [file aem.00495-26-s0001.docx]

Supplementary data

**RegB/A-Dependent Redox Regulation Links Iron Uptake to Methanol Metabolism in *Methylomonas* sp. DH-1**

Wooyoung Park^1#^, Seungwoo Cha^1#^, Hyeonwoo Mun^1^, Kang-Lok Lee^2^, Sang Woo Seo^1^ and Ji-Sook Hahn^1*^

^1^Department of Chemical and Biological Engineering, Institute of Chemical Processes, Seoul National University, 1 Gwanak-ro, Gwanak-gu, Seoul 08826, Republic of Korea

^2^Department of Biology Education, IALS, Gyeongsang National University, JinJu 52828, Republic of Korea

Running Title: Redox Regulation of Iron Uptake in Methanol Metabolism

# These authors contributed equally to this work

^*^Corresponding author:

Ji-Sook Hahn

Phone: +82-2-880-9228

Fax: +82-2-888-1604

e-mail: hahnjs@snu.ac.kr

**Supplementary Tables**

**Table S1** Strains used in this study

| Strain | Description | Genotype | Reference |
| --- | --- | --- | --- |
| *Methylomonas* sp. DH-1 | Wild type strain |  | (1) |
| *∆regA* | *regA* deletion in DH-1 | DH-1 *ΔregA::kan^R^* | This study |
| *∆tbdr* | *tbdr* deletion in DH-1 | DH-1 *Δtbdr::kan^R^* | This study |
| *∆tonB operon* | *tonB* operon deletion in DH-1 | DH-1 *ΔtonB operon::kan^R^* | This study |
| *∆regA-feoO.E.* | *feo* operon overexpression in *∆regA* | DH-1 *ΔregA* *ΔfliE::*P*_mxaF_*-*feoABC*-T*_rrnB_-amp^R^* | This study |
| DH-1 RegA^T7-tagged^ | *T7-regA* overexpression in DH-1 | DH-1 *ΔfliE::*P*_mxaF_*-*T7-regA*-T*_rrnB_-kan^R^* | This study |
| *∆regB/A* | *regB/A* operon deletion in DH-1 | DH-1 *ΔregB/A::amp^R^* | This study |
| DH-1 RegB/RegA | *regB/A operon* expression in *∆regB/A* | *∆regB/A ΔfliE::*P*_regB/A_*-*regB-regA*-T*_rrnB_-kan^R^* | This study |
| DH-1 RegB^H239A^/RegA | H239A mutation on RegB in DH-1 RegB/RegA | *∆regB/A ΔfliE::*P*_regB/A_*-*regB^H239A^-regA*-T*_rrnB_-kan^R^* | This study |
| DH-1 RegB^∆UBD^/RegA | Deletion of ubiquinone binding site on RegB in DH-1 RegB/RegA | *∆regB/A ΔfliE::*P*_regB/A_*-*regB*^∆UBD^*-regA*-T*_rrnB_-kan^R^* | This study |
| DH-1 RegB/RegA^D59A^ | D59A mutation on RegA in DH-1 RegB/RegA | *∆regB/A ΔfliE::*P*_regB/A_*-*regB-regA^D59A^*-T*_rrnB_-kan^R^* | This study |
| *∆Na^+^-nqr operon* | *Na^+^-nqr* operon deletion in DH-1 | DH-1 *Δ Na^+^-nqr operon::kan^R^* | This study |
| *∆AYM39_16990* | *AYM39_16990* deletion in DH-1 | DH-1 *ΔAYM39_16990::kan^R^* | This study |

**Table S2** Recipe of the stock solutions of NMS

| Ingredient | Amount |
| --- | --- |
| 1000X trace element solution | |
| FeSO_4_·7H_2_O | 500 mg/L |
| ZnSO_4_·7H_2_O | 400 mg/L |
| MnCl_2_·7H_2_O | 20 mg/L |
| CoCl_2_·6H_2_O | 50 mg/L |
| NiCl_2_·6H_2_O | 10 mg/L |
| H_3_BO_3_ | 15 mg/L |
| EDTA | 250 mg/L |
| 100X vitamin stock | |
| Biotin | 2.0 mg/L |
| Folic acid | 2.0 mg/L |
| Thiamine HCl | 5.0 mg/L |
| Ca pantothenate | 5.0 mg/L |
| Vitamin B12 | 0.1 mg/L |
| Riboflavin | 5.0 mg/L |
| Nicotinamide | 5.0 mg/L |
| 100X phosphate stock solution | |
| KH_2_PO_4_ | 26 g/L |
| Na_2_HPO_4_ | 32.8 g/L |

**Table S3** Plasmids used in this study

| Plasmid | Description | Reference |
| --- | --- | --- |
| pIns | Plasmid containing [U*_ins_*-T*_rrnB_*-*kan^R^*-D*_ins_*] cassette for integration into noncoding region between *AYM39_05845* and *AYM39_05850* | (2) |
| pFliE-mxaF | Plasmid containing [U*_fliE_*-T*_rrnB_*-[P*_mxaF_*-T*_rrnB_*-*kan^R^*]-D*_fliE_*] cassette for gene integration into *fliE* region and gene overexpression | (3) |
| pIns-regA_Del | pIns-[U*_regA_*-T*_rrnB_*-*kan^R^*-D*_regA_*] | This study |
| pIns-tbdr_Del | pIns-[U*_tbdr_*-T*_rrnB_*-*kan^R^*-D*_tbdr_*] | This study |
| pIns-tonB_Del | pIns-[U*_AYM39_03230_*-T*_rrnB_*-*kan^R^*-D*_AYM39_03210_*] | This study |
| pIns-Na^+^-nqr_Del | pIns-[U*_nqrA_*-T*_rrnB_*-*kan^R^*-D*_nqrM_*] | This study |
| pIns-regB/A_Del | pIns-[U*_regB_*-T*_rrnB_*-*amp^R^*-D*_regA_*] | This study |
| pIns-AYM39_16990_Del | pIns-[U*_AYM39_16990_*-T*_rrnB_*-*kan^R^*-D*_AYM39_16990_*] | This study |
| pFliE-feoABC | pFliE-mxaF-[U*_fliE_*-T*_rrnB_*-[P*_mxaF_*-*feoA-feoB-feoC operon*-T*_rrnB_*-*amp^R^*]-D*_fliE_*] | This study |
| pFliE-T7-regA | pFliE-mxaF-[U*_fliE_*-T*_rrnB_*-[P*_mxaF_*-*T7-G4S-regA*-T*_rrnB_*-*kan^R^*]-D*_fliE_*] | This study |
| pFliE-regB/A | pFliE-mxaF-[U*_fliE_*-T*_rrnB_*-[P*_regB_*-*regB-regA*-T*_rrnB_*-*kan^R^*]-D*_fliE_*] | This study |
| pFliE-regB^∆UBD^/A | pFliE-mxaF-[U*_fliE_*-T*_rrnB_*-[P*_regB_*-*regB*^∆UBD^*-regA*-T*_rrnB_*-*kan^R^*]-D*_fliE_*] | This study |
| pFliE-regB^H239A^/A | pFliE-mxaF-[U*_fliE_*-T*_rrnB_*-[P*_regB_*-*regB*^H239A^*-regA*-T*_rrnB_*-*kan^R^*]-D*_fliE_*] | This study |
| pFliE-regB/A^D59A^ | pFliE-mxaF-[U*_fliE_*-T*_rrnB_*-[P*_regB_*-*regB-regA*^D59A^-T*_rrnB_*-*kan^R^*]-D*_fliE_*] | This study |

**Table S4** Primers used in this study

| Forward primer | Reverse primer | Usage |
| --- | --- | --- |
| Primer sequence for plasmid generation for genetic manipulation in *Methylomonas* sp. DH-1 | | |
| agctGCGGCCGCGTGGAACATGGTGATCATAACTTCGTG | actACTAGTGGATACTCTCCTTAGCTAACAACG | Cloning [U*_regA_*] fragment of pIns-regA_Del with *Not*I*/Bcu*I*,* |
| actGGGCCCCGGGTAATCAGAGGGGGAGCTTGAA | gcgGAGCTCGATTTGGTTGCCTCTAAATAGACCGATTC | Cloning [D*_regA_*] fragment of pIns-regA_Del, pIns-RegB/A_Del with *Apa*I*/Sac*I |
| actGCGGCCGCTTGCCGACAGAATGGCGATG | gcgACTAGTACGTTCCCCTAAGTTGGCTT | Cloning [U*_tbdr_*] fragment of pIns-tbdr_Del with *Not*I*/Bcu*I*,* |
| actGGGCCCCCTTGCCGGGAAACGCCGAA | actGAGCTCCTGCCGAGAGGAACAGCGCGTTT | Cloning [D*_tbdr_*] fragment of pIns-tbdr_Del with *Apa*I*/Sac*I |
| gcgATTTAAATATTGATCTGCATCTAAGTTTCCTTTC | gcgACTAGTAGAGACCTCGTTGTCAGGGT | Cloning [U*_AYM39_03230_*] fragment of pIns-tonB_Del with *Smi*I*/Bcu*I*,* |
| actGGGCCCTTCGCTGATTTCCGGCCTTT | gcgGAGCTCAGTCGACTTGAGTTCCGCTG | Cloning [D*_AYM39_03210_*] fragment of pIns-tonB_Del with *Apa*I*/Sac*I |
| actGCGGCCGCTTATACCTCCATAGTCACGGGAC | gcgACTAGTAGTTAATACGCCTGCTCTCC | Cloning [U*_nqrA_*] fragment of pIns-Na^+^-nqr_Del with *Not*I*/Bcu*I*,* |
| actGGGCCCGGTGCGGGTAGAAGAGTTTAG | gcgGAGCTCCATTAATCGACAAATCGACGATTCC | Cloning [D*_nqrM_*] fragment of pIns-Na^+^-nqr_Del with *Apa*I*/Sac*I |
| actGCGGCCGCGGCTGGCGATTGTCGCCGCGAA | gcgACTAGTGGAAAACCTGCCAGACTTGAATGGG | Cloning [U*_regB_*] fragment of pIns-regB/A_Del with *Not*I*/Bcu*I*,* |
| actGCGGCCGCGTAGGGCAATTCTTCGATATTGCCC | gcgACTAGTATAACAACCTCCAAACGATTTTGG | Cloning [U*_AYM39_16990_*] fragment of pIns-AYM39_16990_Del with *Not*I*/Bcu*I |
| actGGGCCCTGCAGTGGGGTTCGGTGAAG | gcgGAGCTCGGCTGCTTCGAAGAAAAAGC | Cloning [D*_AYM39_16990_*] fragment of pIns-AYM39_16990_Del with *Apa*I*/Sac*I |
| ACTGGGCCCGCGGAACCCCTATTTGT | gcgTTAATTAATCAAGAAGATCCTTTGATCttttc | Exchanging marker from *kan^R^* to *amp^R^* to make pIns-regB/A_Del and pFliE-feoABC |
| gcgGGATCCATGCAGACAAACTTTAAAGC | gcgACTAGTTTAATCCACCCATTCATAGATTTCGG | Cloning [*feoA-feoB-feoC*] fragment of pFliE-feoABC with *BamH*I*/Bcu*I |
| gcgGGATCCATGGCTAGCATGACTGGTGGACAGCAAATGGGTggcgggggtggttctATGACCAATTTGCCTACGGA | gcgACTAGTTCATTCCTTGACCGGTTTTTTCTCCAGC | Cloning [*T7-G4S-regA*] fragment of pFliE-T7-regA with *BamH*I*/Bcu*I |
| actGGCGCGCCGCAGGCGTTATCGAAAGAAATGGCC | gcgACTAGTTCATTCCTTGACCGGTTTTTTCTCCAGC | Cloning [P*_regB_*-*regB-regA*] fragment of pFliE-regB/A with *Sgs*I*/Bcu*I |
| ACGTGCTGGCGTTGGCTTATACGGCGATCATGTTGCCCCA | TGGGGCAACATGATCGCCGTATAAGCCAACGCCAGCACGT | Deleting ubiquinone binding site on pFliE-regB/A to make pFliE-regB^∆UBD^/A |
| GCGGCTgcCGATATGGGCACGCCG | CATATCGgcAGCCGCGCTGGCCGC | Adopting H239A mutation on pFliE-regB/A to make pFliE-regB^H239A^/A |
| GGTAATCGcgTTGCGGATCGGTTTC | ATCCGCAAcgCGATTACCGCGTACT | Adopting D59A mutation on pFliE-regB/A to make pFliE-regB/A^D59A^ |
| Primer sequence for qRT-PCR | | |
| TGGAAGGCAAACAGGCCAAT | GTACTCTATGCTCTTGTCGC | Normalization during qRT-PCR analysis using the *glgA* gene |
| AAGCCGCCGATCCCGACCAAGAA | GCGATTCGATCAATTCTTCCTTGACC | Calculating the expression level of *tbdr* gene (*AYM39_03125*) |
| ATCGCAAGTGGAATTGACCC | TCCTTGCCAATGCATCAGTAG | Calculating the expression level of *AYM39_03230* gene |
| CTACAAGCCATCGCCGTAGCAA | ATTGACCATGATGGCCTGCG | Calculating the expression level of *tonB* gene (*AYM39_03220*) |
| TAAAGCATTGGCGGCTGGCG | GCACCGACGCTTCGTTTTTG | Calculating the expression level of *feoA* gene (*AYM39_11025*) |
| Primer sequence for ChIP | | |
| TGGAAGGCAAACAGGCCAAT | GTACTCTATGCTCTTGTCGC | Negative control primers (*glgA* ORF) |
| TTGAGAACGATTCCAGCAACGCT | ACGGAGTTGTTTGGTGTCGGACA | *tbdr* promoter |
| CATTTGTATCAAATGCCGTTGCTTCTCT | CCGCGGTTTCGGCTTCGTCC | *tonB operon* promoter |
| CCCGCCTGGCTCTGCGATTTTC | GCCAGCCGCCAATGCTTTAAAG | *feo operon* promoter |

**Supplementary Figures**

**
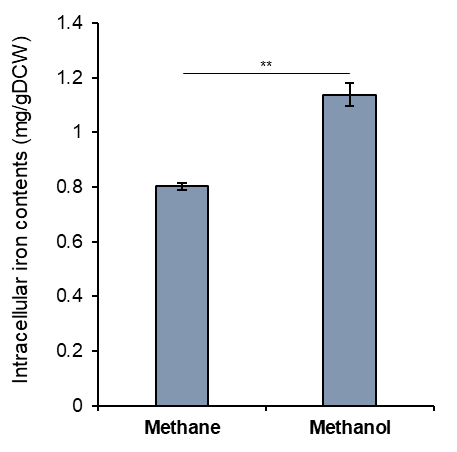
**

**Figure S1.** Intracellular iron content of *Methylomonas* sp. DH-1 under methane- and methanol-grown conditions. 20% (v/v) methane was used for methane feeding and 0.4% methanol was used for methanol feeding. Error bars represent standard deviation (s.d.) from two biological replicates (n = 2). Statistical significance was determined by a two-tailed Student’s t-test (***p*<0.01).

**
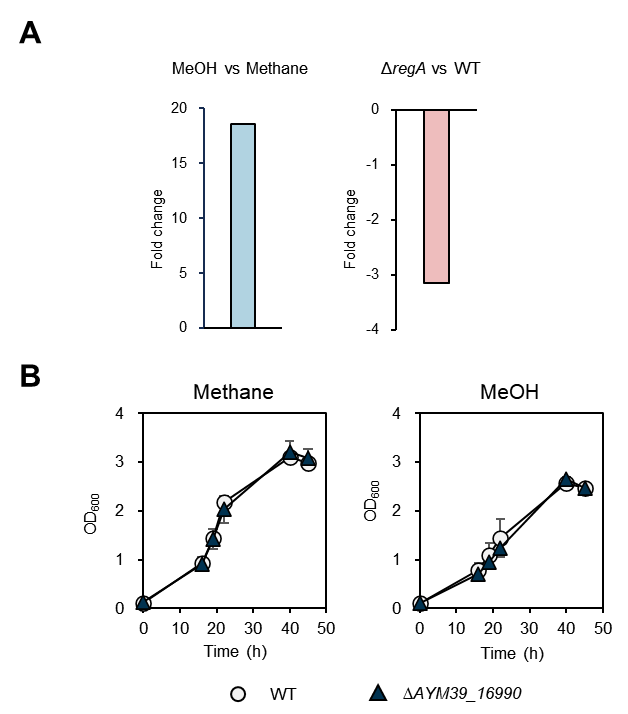
**

**Figure S2. (A)** Expression of *AYM39_16990* was upregulated in *Methylomonas* sp. DH-1 during methanol growth (left panel). Under methanol culture conditions, the expression level of *AYM39_16990* was lower in the *ΔregA* strain than in the wild-type strain (right panel). **(B)** Growth curves of the indicated strains cultured in NMS media with different carbon sources. 20% (v/v) methane was used for methane feeding and 0.4% methanol was used for methanol feeding. Error bars represent standard deviation (s.d.) from three biological replicates.

**
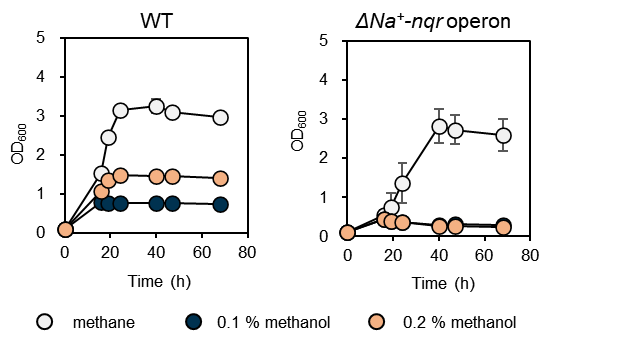
**

**Figure S3.** Growth curves of wild type and *ΔNa^+^-nqr operon* cultured in NMS media with different carbon sources. 20% (v/v) methane was used for methane feeding and 0.1% or 0.2% methanol were used for methanol feeding. Error bars represent standard deviation (s.d.) from three biological replicates.

**References**

1. Hur DH, Na J-G, Lee EY. 2017. Highly efficient bioconversion of methane to methanol using a novel type I Methylomonas sp. DH-1 newly isolated from brewery waste sludge. Journal of Chemical Technology & Biotechnology 92:311-318.

2. Lee JK, Kim S, Kim W, Kim S, Cha S, Moon H, Hur DH, Kim S-Y, Na J-G, Lee JW, Lee EY, Hahn J-S. 2019. Efficient production of d-lactate from methane in a lactate-tolerant strain of Methylomonas sp. DH-1 generated by adaptive laboratory evolution. Biotechnology for Biofuels 12:234.

3. Cha S, Cho Y-J, Lee JK, Hahn J-S. 2023. Regulation of acetate tolerance by small ORF-encoded polypeptides modulating efflux pump specificity in Methylomonas sp. DH-1. Biotechnology for Biofuels and Bioproducts 16:114.
